# 

**Published:** 1896-02-15

**Authors:** 


### EARLY EDITION.

**Notices of Births, Deaths, and Marriages** are inserted in this column at a charge of 2s. 6d. for an announcement not exceeding four lines.

**Notice to Advertisers and Subscribers.**

All Advertisements, Orders for Copies of The Hospital and Business Communications should be addressed to **THE MANAGER, (Not to The Editor)**, The Hospital, 428, Strand, London, W.C.

The Cost of Subscription, post free, is as follows:—Per week, 2½d.; per quarter, 2s. 8d.; per half-year, 5s. 3d.; per annum, 10s. 6d. Foreign:—Per Annum, 15s. 2d.; payable, in all cases, in advance.

### NOTICE TO CORRESPONDENTS.

All MS., Letters, Books for Review, and other matters intended for the Editor should be addressed The Editor, The Lodge, Porchester Square, London, W.

The Editor cannot undertake to return rejected MS., even when accompanied by stamped directed envelope.

**"Burdett's Hospitals and Charities."**

Sixth year of publication. 950 pp. Scarlet cloth, gilt lettered. Price 5s. A most complete guide to British, Colonial, Indian, and American Hospitals, Dispensaries, Nursing and Convalescent Institutions, and Asylums. A few complete sets of the preceding five issues of the "Annual" may still be obtained on application to the Publishers, The Scientific Press (Limited), 428, Strand, London, W.C.

**NOTICE.—Vol. XVIII. of "The Hospital" (April to September, 1895), in handsome cloth binding, is on sale at the Office of this Journal. Price 7s. 6d. Cases for binding the Numbers contained in this Volume, 1s. 6d. Index to Vol. XVIII., 2½d., post free.**

**The Monthly Part for February is now ready, price 6d., by post, 8d.**

### SCRAPS AND GLEANINGS.

IT is proposed to provide a cottage hospital for Malton, Kent.

MR. EDWARD WHITE, a member of the Brighton Town Council, has contributed £1,000 to Brighton charities in commemoration of his 60th birthday.

IN our recent notice on the Derbyshire Royal Infirmary we quoted the income as £5,218. This sum, however, represents only subscriptions, donations, and offertories. The total ordinary income for the past year was £7,356.

THE Lenzie Convalescent Home, Glasgow, whilst presenting a good account of the last 12 months' work, shows a deficit on the accounts of £162.

THE Merchant Taylors' Company have sent a donation of £10 10s. to the building fund of the new children's branch at Broadstairs of the Metropolitan Convalescent Institution.

THE chairman of the Royal Hospital for Incurables at Dublin has contributed £100 towards claiming the offer of a similar amount made by Mr. Marcus Tertius Moses, the donation to qualify four new life governors for the institution.

THE designs for the erection of new fever hospitals for the City of Leeds have now formally received the sanction of the Local Government Board.

AMONG the many liberal donations received during the year by the Midland Counties Home for Incurables, mention is made of £200 from the late Mr. G. C. Benn, of Rugby, given in memory of his brother. The subscriptions and donations have also increased considerably.

THE Norfolk and Norwich Eye Infirmary has benefited to the extent of £100 from the late Mr. Fitch.

THE annual smoking concert promoted by the Nottingham and Notts Hospital Saturday Committee has taken place.

THE net proceeds of the dance in favour of the funds of the Drumcondra Hospital, Dublin, brought in £32.

THE falling off of subscriptions at the Worcester Ophthalmic Hospital is a matter to be much regretted. The committee are considering ways and means of improving this condition of affairs.

THE celebrated collection of "Jenner Relics," got together by Mr. Frederick Mockler, of Wotton-under-Edge, which were exhibited in 1893 at the Bristol Exhibition, will find a permanent home at University College, Bristol, provided that sufficient public subscriptions be got together for their purchase.

### BOOKS RECEIVED.

KEGAN PAUL AND TRENCH.

"The Art of Compounding." By Wilbur L. Scoville, Ph.G.

The GARDENERS' MAGAZINE Office.

"The Garden Oracle and Floricultural Yearbook for 1896."

W. G. MOORE AND CO., Birmingham.

"Temperance or Total Abstinence?" By Frederick Baker, A.C.P.

JOHN WRIGHT AND CO., Bristol; SIMPKIN AND CO., London.

"A Pharmacopœia for Diseases of the Skin." By James Startin.

**Periodicals and Pamphlets.**—Cassell's History of England, New York Medical Journal, Clinical Sketches, Literary Digest, London, Clinical Journal, Invention, Cassell's Family Magazine, Medical Week, Ladies' Gazette, Columbus Medical Journal, Zenana, British Battles on Land and Sea, Sanitary Journal, Cornhill Magazine, Medical Press, Erin.

### The Student of Medicine.

### APPOINTMENTS.

C. H. Allfrey, M.D.Edin., M.R.C.P.Lond., F.R.C.S.Eng., D.P.H.Camb., appointed Assistant Physician to the Hastings, St. Leonard's, and East Sussex Hospital. E. Annacker, M.D.Berlin, M.R.C.S., appointed Honorary Physician to the Hulme Dispensary. C. Atkin, F.R.C.S.Eng., appointed Surgeon to the Sheffield General Infirmary. H. H. Back, M.B.Lond., M.R.C.S., appointed Medical Officer of Health to the Aylsham District Council. Dr. J. Blackford, appointed Medical Officer for the Cradley District of the Stourbridge Union. C. G. Brodie, F.R.C.S.Eng., appointed Assistant Surgeon to the City Orthopædic Hospital. Dr. Brimacombe, appointed Medical Officer for the Oldland District of the Keynsham Union. J. Colville, B.A., M.D.R.U.I., appointed Anæsthetist to the Ulster Hospital for Children and Women, Belfast. J. W. Cook, M.D., M.R.C.S., appointed Medical Officer of Health to the Walton-on-the-Naze Urban District Council. A. R. Darley, M.D., B.Ch., appointed Medical Officer of Health for the Daventry Rural District. F. J. Dixon, M.A., M.D., appointed Assistant Surgeon to the Central London Throat and Ear Hospital. T. D. Forbes, M.B., C.M.Edin., appointed Assistant House Surgeon to the Royal Albert Hospital and Eye Infirmary, Devonport. J. Hamilton, L.R.C.P., L.R.C.S.Edin., reappointed Medical Officer of Health to the Dartford Urban District Council. W. R. Hanbury, L.R.C.P., M.R.C.S., appointed Second Assistant Medical Officer to the County Asylum, Dorchester. Dr. F. H. Hardman, appointed Medical Officer for the Knighton District of the Knighton Union. G. Hardyman, M.B., C.M.Edin., appointed Honorary Surgeon to the Royal Mineral Water Hospital, Bath. J. Harper, L.R.C.P.Lond., M.R.C.S., reappointed Medical Officer of Health to the Padstow Urban District Council. C. J. Heath, appointed Assistant Surgeon to the Central London Throat and Ear Hospital. J. S. Holden, M.D., appointed Medical Officer of Health to the Belchamp and Melford Rural District Council. H. Hoole, M.D.Lond., appointed Medical Officer to the Royal Insurance Company. Dr. C. R. Leighton, appointed Medical Officer for the Skenfrith District of the Monmouth Union. M. J. Madden, L.R.C.P., L.M., L.R.C.S.I., appointed Medical Officer of the Golden Dispensary District of the Tipperary Union. W. J. C. Nourse, F.R.C.S.E., appointed Assistant Surgeon to the Central London Throat and Ear Hospital. E. S. Pollock, B.A., M.B.Dubl., appointed Medical Officer for the Witheridge District of the Southmolton Union. St. George Reid, M.R.C.S., appointed to take charge of the Bacteriological Department of the Central London Throat and Ear Hospital. W. W. Sinclair, M.B.Aberd., appointed Honorary Ophthalmic Surgeon to the East Suffolk and Ipswich Hospital. T. A. Somerville, L.R.C.P.Edin., L.M., M.R.C.S., appointed Medical Officer of Health to the Wilmslow Urban District Council.

### VACANCIES.

#### Resident Medical Officers.

Dr. Steevens's Hospital, Dublin.—Gynæcologist. Applications to the Governers and Guardians of Dr. Steevens's Hospital by February 20th.

Cardiff Union.—Assistant Medical Officer for the Workhouse. Appointment for one year. Salary £100 per annum, with rations, apartments, attendance, and washing. Applications to Arthur J. Harris Clerk, Queen's Chambers, Cardiff, by February 18th.

Chichester Infirmary.—House Surgeon. Salary £80 per annum, with board, lodging, and washing. Applications to the Secretary by February 17th.

Cumberland Infirmary, Carlisle.—Assistant House Surgeon. Appointment for one year. Salary £40 per annum, with board, lodging, and washing. Applications to the Secretary by February 25th.

East London Hospital for Children, Glamis Road, Shadwell, E.—House Physician; must be duly qualified. Board, Lodging, &c., provided, but no salary. Applications to Thomas Hayes, Secretary, by February 29th.

Essex County Asylum, Brentwood. Third Assistant Medical Officer and Pathologist, under 30 years of age. Salary £120 per annum, with board, residence and washing. Applications to the Medical Superintendent by February 15th.

#### Non-Resident Medical Officers.

Dental Hospital of London, Leicester Square.—Assistant Dental Surgeon. Applications to J. Francis Pink, Secretary, by March 9th.

Liverpool Royal Infirmary.—Assistant Honorary Physician. Applications to the Chairman of the Committee of the Royal Infirmary, Liverpool, by February 20th.

London Hospital, Whitechapel, E.—Two Surgeon Dentists. Applications to the House Governor by February 28th.

Middlesex Hospital Medical School.—Lecturer on Anatomy and a Lecturer on Physiology. Applications to the Dean of the Medical School, Cleveland Street, W., by February 17th.

Owens College, Manchester.—Junior Demonstratorship in Physiology and Histology. Salary £100 per annum. Applications to the Registrar by March 2nd.

St. Mark's Hospital, City Road, E.C.—Assistant Surgeon; must be Fellow of the Royal College of Surgeons. Applications to the Secretary, Mr. Arthur Leard, by February 15th.

Westminster Hospital, Broad Sanctuary, S.W.—Surgeon; must be F.R.C.S.Eng. Candidates will be required to attend the House Committee with their testimonials on Tuesday, February 18th, at one o'clock.

### UNIVERSITIES AND COLLEGES.

#### Society of Apothecaries of London.

Pass List, January, 1896.

The following candidates passed in

Surgery.—W. Benton, Charing Cross Hospital; W. Hampson, St. Bartholomew's Hospital; E. W. Herrington, St. Mary's Hospital; and H. E. Wise, Middlesex Hospital.

Medicine, Forensic Medicine, and Midwifery.—E. E. Cornaby; Cambridge, and London Hospital; J. H. Crawshaw, Leeds; W. Hampson, St. Bartholomew's Hospital; M. M. Townsend, King's College, and H. E. Wise, Middlesex Hospital.

Medicine and Midwifery.—F. C. Collinson, Leeds.

Medicine.—E. G. Adams, Royal Free Hospital; H. W. Silver, Charing Cross Hospital; and W. S. Webb, London Hospital.

Forensic Medicine and Midwifery.—T. G. King, London Hospital; W. McCall, Charing Cross Hospital; C. H. Maskew, Birmingham; and H. C. Wimble, St. Bartholomew's Hospital.

Forensic Medicine.—M. Orange, Royal Free Hospital.

Midwifery.—F. R. M. Heggs, Birmingham; and G. S. Taylor, Manchester.

To Messrs. Benton, Cornaby, Hampson, Silver, Townshend, Webb, Wise, and Miss Adams was granted the diploma of the society.

Demy 8vo., profusely Illustrated with Specimen Tables, Index of Classifications, Forms of Tender, &c., cloth extra. Price 6s. (post free).

**THE UNIFORM SYSTEM OF ACCOUNTS, AUDITS, AND TENDERS.**
For Hospitals and Institutions.
By HENRY C. BURDETT.

**ACCOUNT BOOKS FOR INSTITUTIONS.**
A complete set of Royal, Foolscap, and Double Foolscap Account Books, ruled in accordance with the Uniform System adopted by the Metropolitan Hospital Sunday Fund. Designed
By HENRY C. BURDETT.
Descriptive price list post free on application.
The Scientific Press (Limited), 428, Strand, W.C.

### METROPOLITAN HOSPITAL SUNDAY FUND.

### THE ASCLEPIAD.

*A Book of Original Research and Observation in the Science, Art, and Literature of Medicine.*

BY

### SIR BENJAMIN WARD RICHARDSON, M.D., F.R.S.

The ASCLEPIAD, published in four parts to make a volume, is forwarded by post to each subscriber as each part appears, price 2s. 6d. per Part (payable in advance), or 12s. per Volume of Four Parts, handsomely bound in cloth, with Index.

The fourth part of Volume XI., No. 44, will contain articles on—

***Angina Pectoris: Its Nature, Cause, and Treatment.***
***Working Class Sanitation.***
***Opuscula Practica.***
***Thomas Young, M.D., F.R.S.*** (With Portrait.)
***Fountains of Medical Science and Art. Causes of Error in Science.***
***Original Research: Experimental and Inductive.*** Physical Research on Nervous Matter. Part IV.
***Pyrophorous Blood.***
***Transmission of Light through Animal Bodies.***
***Contemporary Practice & Literature.***

LONGMANS, GREEN, AND CO.

### THE BRISTOL MEDICO-CHIRURGICAL JOURNAL.

*Further Permanent Enlargement without increase of price.*

**Published Quarterly, Price 1s. 6d. Annual Subscription, post free, 5s.**

EDITOR—

**R. SHINGLETON SMITH, M.D., B.Sc., F.R.C.P.,** Physician to the Bristol Royal Infirmary, and Emeritus Professor of Medicine in University College, Bristol. With whom are associated—

J. MICHELL CLARKE, M.A., M.D., M.R.C.P., Physician to the Bristol General Hospital, and Professor of Pathology University College, Bristol.

F. R. CROSS, M.B., F.R.C.S., Ophthalmic Surgeon to the Bristol Royal Infirmary, and Surgeon to the Bristol Eye Hospital.

W. H. HARSANT, F.R.C.S., Surgeon to the Bristol Royal Infirmary.

L. M. GRIFFITHS, M.R.C.S., *Assistant Editor*, Hon. Librarian to the Medical Library, University College, Bristol.

B. M. H. ROGERS, B.A., M.D., B.Ch., *Editorial Secretary*, Physician and Pathologist to the Hospital for Sick Children and Women, Bristol.

Books for Review, Exchange Journals, Articles for Notice in the Section of "Notes on Preparations for the Sick," should be sent to the Assistant Editor, L. M. GRIFFITHS, 9, Gordon Road, Bristol. Subscribers Names and Orders for Advertisements to be sent to B. ROGERS, M.D., Editorial Secretary, 11, York Place, Bristol.

**Bristol: J. W. ARROWSMITH. London: J. & A. CHURCHILL.**

### THE QUARTERLY MEDICAL JOURNAL
### FOR YORKSHIRE AND ADJOINING COUNTIES.

EDITED FOR THE PROMOTERS BY
**SIMEON SNELL, F.R.C.S., Ed., &c.**

ASSISTANT EDITORS—

D. BURGESS, M.A., M.B.Cantab.

W. T. COCKING, M.D.Lond.

W. DALE JAMES, M.R.C.S.

ARTHUR J. HALL, B.A., M.B.Cantab.

In association with—

A. W. MAYO ROBSON, F.R.C.S., Leeds.

P. BOOBBYER, M.B., M.S.Durh., Nottingham.

J. A. SOUTHERN, L.R.C.P.Lond., &c., Derby.

PRIESTLEY LEECH, M.D., B.S.Lond., F.R.C.S., Halifax.

W. L. W. MARSHALL, M.R.C.S., Huddersfield.

B. A. WHITELEGGE, B.Sc., M.D.Lond., Wakefield.

W. H. JALLAND, F.R.C.S., York.

E. H. HOWLETT, F.R.C.S., Hull.

ADOLPH BRONNER, M.D., M.R.C.S., Bradford.

E. MANSEL SYMPSON, M.A., M.D. Cantab., Lincoln.

**Sheffield: PAWSON and BRAILSFORD.**

EDINBURGH AND LONDON: YOUNG J. PENTLAND. LONDON: JOHN BALE AND SONS.

**Annual Subscription, 6s. 6d. Single Copies, 2s. each.**

THE 3/6 SERIES OF NURSING MANUALS.

**NURSING: ITS THEORY AND PRACTICE.** Being a complete Text-Book of Medical, Surgical, and Monthly Nursing. By PERCY G. LEWIS, M.D., M.R.C.S., L.S.A. Always a standard work on Nursing, it now occupies the position of a classic, being used throughout the world in Training Schools and by Private Students. Eighth Edition. Greatly enlarged and thoroughly revised. Profusely Illustrated. Crown 8vo, cloth, gilt.

"We warmly recommend it as a nursing manual . . . full of useful hints and information."—*Birmingham Medical Review.*

"Full of interest and instruction . . . cannot fail to assist nurses in their work." —*British Medical Journal.*

**DIET IN SICKNESS AND IN HEALTH.** By MRS. ERNEST HART, formerly Student of the Faculty of Medicine of Paris, and of the London School of Medicine for Women. Demy 8vo, blue buckram, gilt, with numerous illustrations.

With an introduction by Sir Henry Thompson, F.R.C.S., M.B. Lond., who says: —"I do not hesitate to express my opinion that the present volume forms a handbook to the subject thus briefly set forth in these few lines, which will not only interest the dietetic student, but offer him, within its modest compass, a more complete epitome thereof than any work which has yet come under my notice."

**OPHTHALMIC NURSING.** By SYDNEY STEPHENSON, M.B., F.R.C.S.E., Ophthalmic Surgeon to the North-Eastern Hospital for Children, Shoreditch, E., &c., &c. This is a valuable contribution to Nursing literature on a subject hitherto never handled with special reference to Nursing. On account of its compact and clear arrangement, and its numerous illustrations, it is specially recommended to the use of the Nursing Profession. Crown 8vo, cloth, 200 pp. Profusely illustrated with Original Drawings, with list of Instruments required, and a Glossary of Terms.

"May very advantageously be studied by nurses and clinical clerks, or dressers, who are about to enter the ophthalmic service of a hospital."—*The Lancet.*

**SURGICAL WARD-WORK AND NURSING.** By ALEXANDER MILES, M.D. (Edin.), C.M., F.R.C.S.E. A practical Manual of Clinical Instruction for Nurses and Students. This book will be found especially useful to beginners, to whom its simple description of elementary principles and treatment in surgery will render it invaluable. Demy 8vo, 200 pp., cloth boards, copiously illustrated.

"Will furnish much-needed guidance to the student and the nurse in the early stages of their apprenticeship."—*Times.*

**ART OF FEEDING THE INVALID.** By a MEDICAL PRACTITIONER and a LADY PROFESSOR OF COOKERY. A popular treatise on the most frequent disorders; with detailed lists of suitable diet and original recipes for daily use. This important work has been undertaken to supply a want long felt by Matrons and the Lady Superintendents in hospitals and institutions of all kinds where invalids are treated, as well as in private households. It is of the greatest value to nurses in private practice. Demy 8vo, cloth gilt, 260 pp.

**OUTLINES OF INSANITY.** A Popular Treatise on the Salient Features of Insanity. By FRANCIS H. WALMSLEY, M.D., Medical Superintendent of the Darenth Asylum, Metropolitan Asylums Board, Member of Council of Medico-Psychological Association. Demy 8vo, cloth extra, 3s. 6d., or popular edition, stitched, 1s. 6d.

A clearly-written manual in which the various features of mental disorder are fully expounded. The author's style is interesting, and the reader's attention is riveted throughout.

"A most useful little book . . . Many of the descriptive chapters in the body of the work are deeply interesting."—*The Medical Magazine.*

London: THE SCIENTIFIC PRESS (Limited), 428, Strand, W.C.

### THE 2/6 SERIES OF NURSING MANUALS.

**HOW TO BECOME A NURSE: AND HOW TO SUCCEED.** A complete guide to the nursing profession for those who wish to become Nurses, and a useful book of Reference for Nurses who have completed their training and seek employment. Compiled by HONNOR MORTEN, Author of "The Nurses' Dictionary," &c. Third and Revised Edition. Demy 8vo, 209 pp., profusely Illustrated with Copyright Portraits and Drawings.

"To those who are frequently appealed to by girls, or young women, as to the steps they should take to become nurses, this book of Miss Morten's must prove a perfect godsend."—*British Medical Journal.*

**THE NURSES' DICTIONARY OF MEDICAL TERMS AND NURSING TREATMENT.** Compiled for the use of Nurses, by HONNOR MORTEN. Containing descriptions of the principal Medical and Nursing Terms and Abbreviations, Instruments, Drugs, Diseases, Accidents, Treatments, Physiological Names, Operations, Foods, Appliances, &c., &c., encountered in the Ward or Sick-room. Third and Revised Edition (25th thousand). Demy 16mo, suitable for the apron pocket, handsomely bound in handsome leather, gilt, price 2s. 6d. net, or in cloth, price 2s.

"Should be at the disposal of every nurse."—*Birmingham Medical Review.*

**HOSPITAL SISTERS AND THEIR DUTIES.** By EVA C. LUCKES, Matron to the London Hospital. The favourable reception accorded to the first two editions of this useful and interesting work, and the regular demand for copies of it, encourage the publishers to place this third edition, greatly improved and enlarged, before the Institutions. The experience and position of its author entitle it to authority, and every Matron, Sister, or Nurse who aspires to become a Sister, should read the advice and information contained in its pages. Third Edition. Enlarged and partly rewritten, crown 8vo, cloth gilt, about 200 pp.

**MENTAL NURSING.** A Text-book specially designed for the instruction of Attendants on the Insane. By WILLIAM HARDING, M.D., M.R.C.P. The want of a complete book for the instruction of Asylum Attendants has been long felt, and it is with confidence that the publishers recommend this work to the managers of all Institutions for the Insane. Second Edition. Enlarged, demy 8vo, profusely illustrated, nearly 200 pp., cloth.

**SPINAL CURVATURE AND AWKWARD DEPORTMENT:** Their Causes and Prevention in Children. By Dr. GEORGE MULLER, Professor of Medicine and Orthopædics, Berlin. English Edition, edited and adapted by RICHARD GREENE, F.R.C.P. Crown 8vo, cloth gilt, with many Plates and Illustrations.

"Dr. Müller's little book is an able essay upon the effect produced by exercise, attitude, and movement upon the growth and development of the body. He further gives directions which any intelligent parent or teacher could carry out with the help of very simple apparatus, showing how to avoid or, in early cases, to cure certain malformations which, in the majority of cases, arise either from the neglect of very simple hygienic rules or from carelessness."—*Daily Chronicle.*

**THE MOTHER'S HELP AND GUIDE TO THE DOMESTIC MANAGEMENT OF CHILDREN.** By P. MURRAY BRAIDWOOD, M.D., formerly Senior Medical Officer to the Wirral Hospital for Sick Children. Crown 8vo, cloth gilt.

"The book is altogether admirable."—*Manchester Courier.*

London: THE SCIENTIFIC PRESS (Limited), 428, STRAND, W.C.

### "THE HOSPITAL"

AN INDEPENDENT JOURNAL OF
**The Medical Sciences**
AND
**Hospital Administration,**
WITH WHICH IS ISSUED WEEKLY
**A SPECIAL**

### SUPPLEMENT FOR NURSES.

PUBLISHED EVERY SATURDAY.
PRICE TWOPENCE.

Matrons, Sisters, or Nurses will find in THE HOSPITAL a chronicle of the latest Nursing News, Practical Articles of the highest value to those who wish to keep themselves abreast with the times, and brightly written articles from Nurses all over the world.

**SUBSCRIPTIONS CAN COMMENCE AT ANY TIME.**

### TERMS OF SUBSCRIPTION.

### WEEKLY ISSUE.

| | Great Britain. | Foreign and Colonial. |
|---|---|---|
| For One Year ... ... | 10s. 6d. ... | 15s. 2d. |
| For Six Months | 5s. 3d. ... | 7s. 7d. |
| For Three Months... | 2s. 8d. ... | 3s. 10d. |

### MONTHLY PARTS.

| | Post Free to any Part of the World. |
|---|---|
| For One Year ... ... | 8s. 6d. |
| For Six Months ... | 4s. 3d. |
| For Three Months ... ... | 2s. 2d. |

Postal Orders and Cheques should be made payable to "The Scientific Press, Limited."

N.B.—In order to prevent delay, all business communications should be addressed to "THE MANAGER" only, and not to any person individually.

### CHANGE OF ADDRESS.

In event of change of address, notice should be forwarded to the Publishers by the Saturday previous to date of publication, and the old address should also be given.

"THE HOSPITAL" can be obtained of all Booksellers and Newsagents in the United Kingdom, and at all the Railway Stalls, of Messrs. W. H. Smith and Son; and also from the following Agents in the Colonies and Abroad :—

PARIS: Librairie Galignani, 224, Rue de Rivoli.
BERLIN: Messrs. A. Asher and Co.
LEIPSIG: Mr. F. A. Brockhaus.
NEW YORK: The International News Co.
MONTREAL: The Montreal News Co.
TORONTO: The Toronto News Co.
MELBOURNE, SYDNEY, ADELAIDE, and NEW ZEALAND: Messrs. Gordon and Gotch.
INDIA: At all the Railway Bookstalls and Agencies of Messrs. A. H. Wheeler and Co.
SOUTH AFRICA: Messrs. Juta, Cape Town.

PUBLISHING OFFICES: 428, STRAND, LONDON, W.C

*In the Press. Price 5s.*

### BURDETT'S OFFICIAL NURSING DIRECTORY

A DIRECTORY, NOT A REGISTER.

The Hospital states: "It is well to bear in mind the difference and distinction which exists between a register and a directory. A register implies rights, and suggests that those who are not upon it are in some way or another inferior beings. But a directory is another matter. A directory gives as rights; insertion in it implies no adherence to any particular party or any particular opinion, and in no way interferes with entry on the register of any association, or the list of any institute, to which the individual may belong. All that a directory professes to do is to give information. The 'Medical Register' is not a mere list of qualified medical men; it is admission to the Register, which itself makes him qualified. For years, perhaps even now, 'Churchill's Medical Directory' contained the names of medical men who, so far as public appointments were concerned, were unqualified; it also contains the names of homœopathists, whom four out of every five medical men will not meet; and it certainly contains the names of many for whose moral character we should not like to vouch. But it gives full and accurate information about them all, and for the sake of that information it is constantly consulted both by the public and the doctors."

What Nurses Need.

"At present there is no Register for the nursing profession giving any legal status. Such a thing may come or it may not; but in either case there is no reason why there should not be a Directory, giving such information about the career of each nurse as both the public and the profession want; information, by the by, which no legal Register would be likely to insert. The 'Medical Register' is a bald and meagre thing compared with the 'Medical Directory.' What Burdett's 'Official Nursing Directory' proposes to do is to supply the want of a directory giving information. It will give no rights, it will imply no partisanship, it will give no guarantee except that what it prints is true, and is derived from official sources."

THE SCIENTIFIC PRESS (Lim.), 428, Strand, London, W.C.

### ROYAL NATIONAL PENSION FUND FOR NURSES.

*President*—**H.R.H. THE PRINCESS OF WALES.**

*Chairman*—WALTER H. BURNS, Esq. *Deputy-Chairman*—HENRY C. BURDETT, Esq.

**PENSIONS. SICKNESS. ACCIDENT.**

### Total Invested Funds, £250,000.

Nurses are invited to join the Fund on account of the substantial and exceptional advantages which it offers them and which *they cannot obtain elsewhere*. The following are the chief points:—

1. **The Fund is Mutual and essentially Co-operative.**
2. **Easy Payment of Premiums.**
   Nurses can pay their premiums monthly or otherwise as best suits their convenience.
3. **The Fund is open to every Nurse.**
   Nurses can assure for Pensions of any amount commencing at any age.
4. **An Investment and Savings Bank.**
   Those entering under the returnable scale can have their premiums returned to them with compound interest, less a small deduction for working expenses. Interest allowed on deposits.
5. **Additions to Pensions.**
   Besides the Pension entered for, substantial additions thereto may be anticipated in the shape of bonuses.
6. **Sickness and Accident Assurance.**
   Policies are issued in connection with Pension policies assuring 10s., 15s., or 20s. a week in cases of incapacity from work through sickness or accident.

**Full particulars to be obtained from THE SECRETARY,**
ROYAL NATIONAL PENSION FUND FOR NURSES,
28, FINSBURY PAVEMENT, LONDON, E.C.

